# Regional clinical practice patterns in reproductive endocrinology: A collaborative transnational pilot survey of in vitro fertilization programs in the Middle East

**DOI:** 10.1186/1743-1050-4-3

**Published:** 2007-08-28

**Authors:** Eric Scott Sills, Hussein S Qublan, Zeev Blumenfeld, Ahmad VT Dizaj, Ariel Revel, Serdar Coskun, Imad Abou Jaoude, Gamal Serour, Mamdoh Eskandar, Mohammad Ali Khalili, Aygul Demirol, Krinos Trokoudes, Pelin Ocal, Abdul Munaf Sultan, Benjamin A Lotto, Adele El-Kareh

**Affiliations:** 1 Reproductive Endocrinology Division, Department of Obstetrics and Gynecology, Vassar Brothers Medical Center, Poughkeepsie, New York USA; 2 Department of Obstetrics and Gynecology, Royal Jordanian Medical Service, Amman, Jordan; 3 Division of Reproductive Endocrinology, Department of Obstetrics and Gynecology, Rambam Medical Center/Technion Faculty of Medicine, Haifa, Israel; 4 Royan Institute, Tehran, Iran; 5 Department of Obstetrics and Gynecology, Hadassah Medical Center and Hebrew University-Hadassah Medical School, Jerusalem, Israel; 6 Department of Obstetrics and Gynecology, King Faisal Specialist Hospital and Research Center, Riyadh, Saudi Arabia; 7 Center for Reproductive Medicine and Genetics, Beirut, Lebanon; 8 Egyptian IVF Center, Al-Azhar University, Cairo, Egypt; 9 Maternity and Child Hospital, The Saudi Center for Assisted Reproduction, King Khalid University College of Medicine, Abha, Saudi Arabia; 10 Research and Clinical Center for Infertility, Shahid Sadoughi University of Medical Sciences, Yazd, Iran; 11 Women's Infertility, IVF and Health Clinic, Ankara, Turkey; 12 Pedieos IVF Center, Nicosia, Cyprus; 13 IVF-ET Unit, Department of Obstetrics and Gynecology, Cerrahpasa Medical Faculty, Istanbul University, Istanbul, Turkey; 14 Assisted Conception Unit, Women's Hospital, Hamad Medical Corporation, Doha, Qatar; 15 Department of Mathematics, Vassar College, Poughkeepsie, New York USA; 16 Department of Obstetrics and Gynecology, Vassar Brothers Medical Center, Poughkeepsie, New York USA

## Abstract

**Background:**

This research describes current clinical and demographic features sampled from reproductive endocrinology programs currently offering in vitro fertilization (IVF) in the Middle East.

**Methods:**

Clinic leadership provided data via questionnaire on patient demographics, demand for IVF services, annual cycle volume, indications for IVF, number of embryos transferred, twinning frequency, local regulations governing range of available adjunct therapies, time interval between initial enrollment and beginning IVF as well as information about other aspects of IVF at each center.

**Results:**

Data were received from representative IVF clinics (*n *= 13) in Cyprus, Egypt, Iran, Israel, Jordan, Lebanon, Qatar, Saudi Arabia and Turkey. Mean (± SD) age of respondents was 47.8 ± 8 yrs, with average tenure at their facility of 11.2 ± 6 yrs. Estimated total number of IVF programs in each nation responding ranged from 1 to 91. All respondents reported individual participation in accredited CME activity within 24 months. 76.9% performed embryo transfers personally; blastocyst transfer was available at 84.6% of centers. PGD was offered at all sites. In this population, male factor infertility accounted for most IVF consultations and the majority (59.1%) of female IVF patients were < 35 yrs of age. Prevalence of smoking among female IVF patients was 7.2%. Average number of embryos transferred was 2.4 (± 0.4) for patients at age < 35 yrs, and 2.9 (± 0.8) at age > 41 yrs. For these age categories, twinning (any type) was observed in 22.6 (± 10.8)% and 13.7 (± 10.4)%, respectively. In 2005, the average number of IVF cycles completed at study sites was 1194 (range 363–3500) and 1266 (range 263–4000) in 2006. Frozen embryo transfers accounted for 17.2% of cycles at these centers in 2005. Average interval between initial enrollment and IVF cycle start was 8 weeks (range 0.3–3.5 months).

**Conclusion:**

This sampling of diverse IVF clinics in the Middle East, believed to be the first of its kind, identified several common factors. Government registry or oversight of clinical IVF practice was limited or nonexistent in most countries, yet number of embryos transferred was nevertheless fairly uniform. Sophisticated reproductive health services in this region are associated with minimal delay (often < 8 weeks) from initial presentation to IVF cycle start. Most Middle East nations do not maintain a comprehensive IVF database, and there is no independent agency to collect transnational data on IVF clinics. Our pilot study demonstrates that IVF programs in the Middle East could contribute voluntarily to collaborative network efforts to share clinical data, improve quality of care, and increase patient access to reproductive services in the region.

## Background

Free exchange of medical information at departmental or multi-institutional levels has been characterized as an important, positive component of modern medical practice [1,2]. Such forums ideally provide opportunities for medical staff to refine diagnostic and management strategies in a collegial, educational setting [3]. In the Middle East, international symposia and conferences can also facilitate these professional goals, yet the current situation there can present potential impediments to dialogue.

As has been the case in other areas of occasional turmoil, political and religious discord in the Middle East has thus far resisted attempts to bring a lasting reconciliation. In the meantime, IVF physicians of the region are daily called upon to see beyond this distraction and are expected to solve complicated, frustrating problems for their patients. An unusual, hopeful symbolism emerged from this background, where providers of the advanced reproductive technologies were considered as being well-placed observers of what can happen when measured combinations of balanced and opposing elements are carefully brought together, at the most basic humanistic level, to yield new, positive beginnings. It was the aim of this pilot study to engage IVF physician leaders from throughout the Middle East in a preliminary, voluntary international discourse on the advanced reproductive technologies.

## Methods

This research sampled data on IVF practice patterns (national, institutional and individual) in the Middle East using data derived from a voluntary 35-part questionnaire, developed in consultation with a multidisciplinary team and influenced by current SART reporting guidelines (USA). It consisted of three sections: 1) general questions about IVF in the sample country, 2) questions about IVF practiced at the respondent clinic, and 3) specific data on the individual completing the questionnaire (Table 1).

**Table 1:** Questionnaire.

**Nation/state queries::**
1) In your country, what is the grand total of IVF centers in operation there at present (defined as a facility currently capable of performing oocyte retrieval and embryo transfer)?
2) In your country, when was the *very first *IVF cycle actually undertaken? If that first cycle did not result in a birth, then in what year was the first IVF cycle that yielded a viable delivery?
3) In your country, is there a national registry of IVF data? If not, is one planned or under discussion?
4) In your country, describe the range of costs typically associated with IVF? Is this expense typically covered by insurance, governement, or other third-party payment?
5) In your country, is donor oocyte IVF lawfully permitted, and if yes, how much are the donors compensated (if anything)?
6) In your country, is IVF regulated or controlled by any national or other governmental agency? If yes, please describe.
7) In your country, are physicians and/or IVF clinics permitted to advertise to the public directly regarding advanced reproductive medical treatment?
8) In your country, is preimplantation genetic diagnosis legally permitted?
9) In the next five (5) years, do you anticipate the number of IVF centers in your country will increase, decrease, or remain essentially unchanged?

**Queries specific to your institute::**
1) When did your facility open (treat first patient for IVF)?
2) At your center, what percentage of your IVF patients are under age 35 at time of their IVF cycle?
3) At your center, what percentage of your IVF patients are age 35–37 at time of their IVF cycle?
4) At your center, what percentage of your IVF patients are age 38–40 at time of their IVF cycle?
5) At your center, what percentage of your IVF patients are over age 41 at time of their IVF cycle?
6) At your center, what is the average number of embryos transferred for each of the 4 age categories above?
7) At your center, what was the total number of IVF patients who had an embryo transfer in 2006? In 2005?
8) Has your center ever performed a blastocyst transfer?
9) At your center, what is the average rate of twin pregnacy (either monozygotic or dizygotic) for each of the 4 age categories above?
10) At your center, what percentage of your IVF patients undergo thawed embryo transfer (FET)?
11) At your center, what percentage of your IVF patients are donor-egg recipients?
12) At your center, what is the most common indication for IVF? What percentage of your IVF cycles are done exclusively for male factor infertility?
13) At your center, what percentage of your IVF patients are smokers (this question concerns female patients only, not partners)?
14) What is the approximate cost (in local currency) for basic IVF (without ICSI, PGD etc) at your center?
15) At your center, what is the approximate interval of time between when an (uncomplicated) IVF patient first registers and when her IVF programme actually starts?
16) At your center, for patients who successfully conceive after IVF, at what gestational age are they usually released from your clinic to return to their OB provider?
17) Does your facility operate any branch or satellite offices to facilitate cycle monitoring for IVF patients? If yes, how many?

**Questions for the person answering this inquiry::**
1) What is your current position with your IVF clinic (lab director, physician, etc)?
2) How long have you worked at your current IVF programme?
3) When was the approximate date (and location) of the last accredited meeting/conference you attended?
4) What is your present age?
5) Have you ever presented your research at an accredited meeting/conference (either oral or poster)?
6) Do you have any academic affiliation? If yes, please describe.
7) Do you personally perform embryo transfers?
8) Do you personally accept appointments for IVF patients (either new or returning)?
9) Do you personally attend patients at births, perform Cesarean deliveries, or otherwise provide obstetric services to patients?

The definition of "Middle East" was a modified version of that used for current international tax calculations and airfare determination as established by the International Air Transport Association (IATA). The IATA lists the following as constituents of the "Middle East": Bahrain, Egypt, Iran, Iraq, Israel, Jordan, Kuwait, Lebanon, Pakistan, Palestinian territories, Oman, Qatar, Saudi Arabia, Somalia, Sudan, Syria, United Arab Emirates, and Yemen. Note that IATA nomenclature includes Pakistan, Somalia and Sudan as Middle East nations, although for our investigation these were omitted and Cyprus and Turkey (IATA included neither as "Middle East") were substituted.

The US National Library of Medicine/PubMed public search engine was used to query each country from this roster coupled with search terms "IVF" or "embryo" to identify clinics currently involved in published fertility research. Abstracts retrieved with author e-mail contact(s) were then used to communicate with each clinic. It was anticipated that some IVF programs might choose not to contribute formal research papers to the medical literature (and thus be missed by Medline/PubMed search). Therefore, general public internet search engines were also queried in the same manner to locate advertisements, press releases, downloadable IVF patient instructions, etc. originating from programs of the region. When neither method identified at least one IVF center within a Middle East country, the American citizen's service office at the local US. Embassy was directly contacted to verify that our search was complete or to confirm that IVF was not currently offered at any site within that country.

Data were received by secure email and tabulated by country of origin; summary data were reported where only one center per country responded. Each facility was given an opportunity to verify all information before final analysis. No unsolicited questionnaires were tabulated for analysis; the Wilcoxon rank-sum test was used for comparison between groups.

## Results

Completed questionnaires were received from 13 IVF programs over a period of four months representing nine nations in the Middle East. Data were provided by two programs in Iran, Israel, Saudi Arabia and Turkey. Basic demographic information on the region stratified by responding and non-responding countries is summarized in Table 2.

**Table 2:** Comparison of demographic features among nations where sample IVF data were collected (a) and non-sampled nations (b).

Country		Population (in millions)	Growth rate (%)	Birth rate (per 1,000)	TFR^1^	Life expectancy^2^	
						
						M	F
Cyprus	a	0.79	0.5	12.6	1.8	75.6	80.5
Egypt	a	80.3	1.7	22.5	2.8	69.0	74.2
Iran	a	65.4	0.7	16.6	1.7	69.1	72.1
Israel	a	6.4	1.2	17.7	2.4	77.4	81.9
Jordan	a	6.1	2.4	20.7	2.6	76.0	81.2
Lebanon	a	3.9	1.2	18.0	1.9	70.7	75.8
Qatar	a	0.9	2.4	15.6	2.8	71.6	76.8
Saudi Arabia	a	27.6*	2.1	29.1	3.9	73.9	78.0
Turkey	a	71.2	1.0	16.4	1.9	70.4	75.5
*subtotal*		262.6					
Bahrain	b	0.7	1.4	17.5	2.6	72.2	77.3
Iraq	b	27.5	2.6	31.4	4.1	68.0	70.1
Kuwait	b	2.5	3.6	22.0	2.9	76.3	78.5
Oman	b	3.2	3.2	35.8	5.7	71.4	76.0
Syria	b	19.3	2.2	27.2	3.3	69.3	72.0
UAE	b	4.4	4.0	16.1	2.4	73.2	78.4
Yemen	b	22.2	3.5	42.7	6.5	60.6	64.5
*subtotal*		79.8					
*p *(a vs. b)^3^			< 0.01	0.09	0.03	0.47	0.30

*Notes*: ^1^TFR=total fertility rate (children born/woman) ^2^at birth, reported in years ^3^by Wilcoxon rank-sum test *includes 5.6 million non-nationals

source: CIA World Factbook (June 2007)

Mean (± SD) age of respondents was 47.8 ± 8 yrs. On average, these individuals reported being at their respective clinics for 11.2 ± 6 yrs. All study participants reported participation in accredited CME meetings within the previous 24 months, and 100% had presented formal research reports before such bodies. Conference sites attended by staff included Aqaba (Jordan), Barcelona (Spain), Cairo (Egypt), Durban (South Africa), Lyon (France), New Orleans (USA), Valencia (Spain), and Yazd (Iran). Most (10/13) personally performed embryo transfer, and 11 of 13 (84.6%) were actively involved in new or returning IVF patient consultation. While all programs offered prenatal referrals outside their clinic by the end of the first trimester, 8 of 13 (61.5%) providers indicated they were involved in obstetrical care for their pregnant IVF patients by "attending them at delivery". None of the sampled IVF programs identified any local ordinances or regulations prohibiting advertisement of advanced reproductive medical services directly to the public; as expected, estimated total number of IVF programs generally varied with population (range 1–91, data not shown).

In this population, most couples presenting for IVF included women < 35 yrs of age (59%), and 8.7% of such female IVF patients were age > 41 (see Table 3). Delivery, clinical pregnancy, or miscarriage rates were not tabulated by institution for this study. We found PGD to be legally available in all sampled countries of the Middle East, and extended in vitro culture permitting blastocyst transfer was reported by 84.6 % (11 of 13) centers (data not shown). Anonymous donor oocyte IVF was offered in Cyprus, Iran, Israel and Lebanon, but only under specified circumstances in the latter three countries. Elsewhere in the Middle East this service was not available. Patient cost for IVF was, on average, under US$2500 and sometimes free (Table 3). Because no contact information was available from any source regarding IVF centers in Bahrain, Iraq, Kuwait, Oman, Palestinian territories, Syria, UAE or Yemen, no data could be obtained from these countries.

**Table 3:** Summary of patient and treatment characteristics from representative IVF centers (*n *= 13) in the Middle East.

		Age (yrs)			
		
		< 35	35–37	38–40	> 41
Patient distribution	%	59.1 (15.6)	17.9 (6.5)	14.1 (9.9)	8.7 (7.2)
Twinning frequency^a^	%	22.6 (10.8)	18.1 (8)	13.7 (10.5)	13.7 (10.4)
FET	%			17.3	
Smoking^b^	%			7.2	
#ET		2.4 (0.4)	2.7 (0.6)	2.8 (0.6)	2.9 (0.9)
Enroll-to-treat interval^c^	Weeks			7.5	
OB referral time^d^	Weeks			8	
% Male factor				50.3	
Direct patient cost/cycle^e^				2500 (range 0–4059)	

*Notes*: Data reported as mean (± SD), except as noted FET=frozen embryo transfers (all ages) ET=total embryo transfers

^a^monozygous + dizygous

^b^reported tobacco use among female patients only (all ages)

^c^average time from first appointment to gonadotropin start for IVF (all ages)

^d^gestational age when patient discharged from IVF clinic to obstetrician for prenatal care (includes programs who discharge patients on the day following ET and those who offer prenatal care in continuity with IVF treatment

^e^mean patient cost/IVF cycle in USD (some programs provide first 2 IVF cycles free to qualified residents)

## Discussion

An area rich in history and tradition, the Middle East is also a land of contemporary economic and political struggle. Indeed, the longstanding geopolitical challenges of the region make stability and safety highly prized–for those outside the region and local residents as well. Against this backdrop of complex and sometimes conflicting interests is a population of > 300 million, where the deeply personal experience of childlessness or miscarriage must still be confronted. When viewed from beyond the Middle East, the individual patient journey with infertility risks being obscured by public headlines of regional unrest. This research sought to focus attention on the advanced reproductive technologies here, with emphasis on establishing collaborative exchange among clinics and practitioners who have made it a priority to assure the service remains available.

Although previous multinational reports on IVF practice have made important contributions to our field [4-6], none have focused specifically on the Middle East. It is an admittedly difficult arena in which to conduct international research, yet the current study finds IVF experts throughout the Middle East willing to share data and work collaboratively. Wherever contacts could be made, institutions throughout the region unconditionally accepted the invitation to participate in this voluntary study.

Recognizing differences across political, religious, or cultural spectrums, our investigation identified common findings not previously noted in a multinational Middle Eastern sample. For example, a relatively high proportion of male factor indications for IVF (with attendant need for ICSI) noted in smaller Middle Eastern groups [7], and the current study; supports extension of this earlier finding to a broader population of the region.

Additionally, compared to Western practitioners, Middle East IVF physicians more often offer seamless care for patients conceiving after IVF with many personally attending their patients at delivery. This is in sharp contrast to USA practice, where the doctor performing embryo transfer is very rarely the same practitioner dividing the umbilical cord at delivery. Differences in patient comfort and satisfaction between the "unified" vs. "fragmented" provider approach have not been specifically studied from the perspective of the medical consumer, but form the basis of further research.

Another characteristic among sampled clinics was the presence of government subsidies for qualified infertility treatment. In many of the countries studied here, national health policy has tended to favor public support of costly IVF programs [8], in contrast to the general experience in USA where public mandates and private insurance coverage for IVF are uncommon [9,10]. Our data were also consistent with those reported previously [11], in that even where expense associated with IVF was not subsidized, average direct cost to IVF patients in these countries remained markedly lower than the cost of comparable treatment in USA. Furthermore, this study found that despite limited or non-existent formal government regulation of clinical IVF practice in the region, the number of embryos transferred remained nevertheless fairly uniform among the sampled institutions (Table 3).

Rapid advancements in the assisted reproductive technologies have frequently brought IVF providers to the forefront of bioethical and religious dialogues concerning which therapies can or should be offered. Muslim beliefs historically have included a proscription against third-party donation in infertility, and IVF is usually regarded as permissible with the proviso that it does not involve use of donor gametes or surrogacy [11-13]. Likewise Judaism allows use of assisted reproduction treatments as long as the oocyte and sperm originate from the couple themselves [14-16]. The clinics sampled here appear to have successfully adapted their mode of practice, conforming to these expectations. Indeed, there was no projected decline in the number of local IVF centers in this geographical region, indicating a population comfortable with present practice standards/outcomes and where demand for the advanced reproductive technologies is likely to grow.

Several limitations of this investigation must be acknowledged. Sampling only one or two clinics per country may not represent an accurate national cross-section of all IVF activity. It was impossible to locate and establish contact with all IVF programs in the Middle East, however. The total number of extant IVF clinics in each nation was essentially an estimate provided by helpful practitioners who may not have access to current, comprehensive statistics. A meaningful "response rate" to our questionnaire therefore could not be calculated. While demographic comparison between the sampled and non-sampled countries found few important differences, measures of population growth rate and total fertility were significantly higher among non-sampled nations. It may be that this finding reflects reduced local demand for IVF clinical services, and may partially explain the difficulty in retrieving data on IVF from these sites.

Descriptive research of this type also invites reporting bias. Since it is possible that some IVF programs in the Middle East received our questionnaire but declined to respond after reviewing its content, we may have unintentionally selected IVF centers that were better organized or with superior statistics. A solution to such ascertainment bias may emerge as governmental agencies gather this type of data more effectively and include a mandate to make it available to the general public. The difficulty in establishing contacts with some IVF facilities in the region implies efforts to improve communication via internet would be helpful, not just for inter-clinic cooperation but also to facilitate patient education and better awareness of services.

## Conclusion

It has never been more critical for IVF providers to be mindful of cultural and religious perspectives on the advanced reproductive technologies, as such social factors often work actively to shape public opinion on complicated bioethical matters. Since clinical outcomes may be compared only if treatment is carried out under equivalent circumstances and complete standardized data are collected, building bridges to share information freely across international borders is crucial. It has been shown that appropriately designed registries contribute to effective assessment in reproductive health technology [17], and this pilot study provides an encouraging framework to achieve this goal in the Middle East.

## Competing interests

The author(s) declare that they have no competing interests.

## Authors' contributions

ESS, HSQ, ZB, AVTD, AR, SC, IAJ, GS, ME, MAK, AD, KT, PO, AMF, BAL and AE provided data and editorial comment; all authors read and approved the final manuscript. BAL provided statistical oversight. ESS and AE conceptualized the project, coordinated drafts and organized the research.
